# Rare Evolutionary Events Support the Phylogenetic Placement of Orthonectida Within Annelida

**DOI:** 10.3390/ijms26135983

**Published:** 2025-06-21

**Authors:** Olga V. Nikolaeva, Kirill V. Mikhailov, Maria S. Muntyan, Oleg A. Zverkov, Sergey A. Spirin, Vassily A. Lyubetsky, Georgy S. Slyusarev, Vladimir V. Aleoshin

**Affiliations:** 1 Belozersky Institute of Physico-Chemical Biology, Lomonosov Moscow State University, Leninskie Gory, 119992 Moscow, Russia; olga_popova92@inbox.ru (O.V.N.); kv.mikhailov@belozersky.msu.ru (K.V.M.); sas@belozersky.msu.ru (S.A.S.); 2 Institute for Information Transmission Problems (Kharkevich Institute), Russian Academy of Sciences, Bolshoy Karetny Per., 19, Bld. 1, 127051 Moscow, Russia; zverkov@iitp.ru (O.A.Z.); lyubetsk@iitp.ru (V.A.L.); 3 Higher School of Economics, Myasnitskaya St., 20, 101000 Moscow, Russia; 4 Department of Invertebrate Zoology, Faculty of Biology, Saint-Petersburg University, Universitetskaya Emb. 7/9, 199034 St. Petersburg, Russia; g.slusarev@spbu.ru; 5 Faculty of Biology, Lomonosov Moscow State University, Leninskie Gory, 119234 Moscow, Russia

**Keywords:** orthonectida, mitogenome, phylogeny, long branch attraction (LBA), synapomorphy

## Abstract

Orthonectids are a group of highly simplified worm-like parasites that are placed within Lophotrochozoa by multigene mitochondrial and nuclear phylogenies. However, their exact position within Lophotrochozoa is uncertain due to the high rate of molecular evolution and putative long branch attraction artifacts. To examine the phylogenetic placement of orthonectids, we applied an alternative approach that takes into account rare evolutionary events (gene order rearrangements in mitochondrial DNA and individual changes in mitochondrial proteins) with an assessment of their probabilities based on a reference sequence database (RefSeq, NCBI). This approach strongly supports the branching of orthonectids among annelids, but does not conclusively resolve their position among the annelid taxa.

## 1. Introduction

Mitochondrial DNA is a ubiquitous element of eukaryotic genomes and a popular marker for phylogenetic studies. As of March 2025, there are 15,885 complete mitochondrial genomes of Metazoa in the NCBI Reference Sequence Database (RefSeq), mostly sequenced for phylogenetic studies. This ample database can be used as a source for complementing traditional tree-building approaches with information on the evolution of genomic features, such as gene order. We decided to examine the controversial problem of phylogenetic placement of Orthonectida using this database as a reference for evaluating the likelihood of emergence and loss of rare characters in the mitochondrial sequences.

Orthonectids are an *incertae sedis* group of spiralians or lophotrochozoans, usually viewed as members of the segmented worms (Annelida). They include about 25 species of obligate parasites of various marine invertebrates [1,2], but their actual diversity is likely underestimated. The orthonectid life cycle includes two alternating generations: (*i*) the parasitic plasmodium, which resides and feeds within the host tissues, producing mitotic internal cells (agametes) that develop into (*ii*) ephemeral nonfeeding females and males, which exit the host’s body to reproduce [2]. In the 19th century, orthonectids were primarily regarded as primitive animals, and together with the cephalopod parasites dicyemids, they were classified as Mesozoa—a supposedly intermediate link between unicellular and multicellular animals. Modern microscopy methods have confirmed the absence of digestive, excretory, circulatory systems and the gonad wall in orthonectids at all life cycle stages, but have identified the presence of nervous and muscular systems in the free-living ephemeral individuals [3,4,5]. Phylogenomic studies have clearly confirmed that orthonectids belong to Lophotrochozoa [6,7,8,9,10], which implies that their simplified morphological organization arose secondarily as a result of parasitic lifestyle. But the exact phylogenetic position of orthonectids remains controversial. While some reconstructions place orthonectids within annelids [8,9,11], other reconstructions revive the hypothesis of a relationship between orthonectids and dicyemids, which are placed within Lophotrochozoa outside annelids [7,10]. This discrepancy persists despite the use of extensive phylogenomic datasets in tree reconstruction. The obvious reason for these contradictions is the extremely high level of sequence divergence in orthonectids and dicyemids, and the ensuing difficulties in tree reconstruction for species with highly uneven evolutionary rates [12,13,14,15,16].

In addition to the various tree reconstruction methods based on the nucleotide or amino acid substitution models that currently dominate the field of phylogenetic inference, phylogenetic signal can also be obtained from rare evolutionary events [17,18,19,20,21,22,23,24]. Such events include deletions, insertions, transpositions, changes in the nucleotide/amino acid composition or protein domain order (protein architecture), and changes in the genetic code. In the current paradigm, such changes are not included in the phylogenetic analysis or are subsumed by the evolutionary models, because there is insufficient data to quantify their “rarity”. Commonly, individual amino acid or nucleotide substitutions are not considered rare events, although, depending on their localization in evolutionarily conserved sites, they can arise as rarely as deletions, insertions, and transpositions. Here we implement the idea of using a large set of mitogenomic data to obtain such estimates and complement the standard approach for phylogenetic inference by quantifying these “rare” events.

## 2. Results and Discussion

### 2.1. Organization of Orthonectid Mitochondrial Genomes

**Figure 1:**
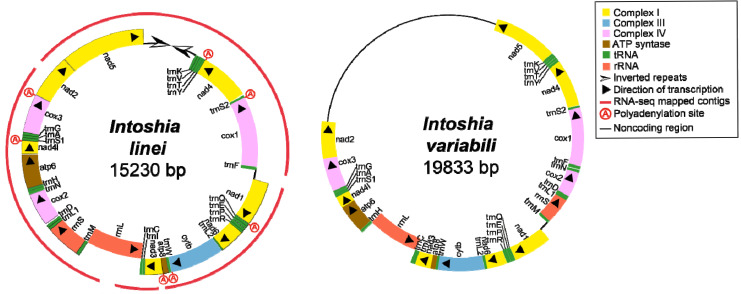
Mitochondrial genomes of *Intoshia linei* and *I. variabili*.

Complete or nearly complete mitochondrial genomes of three orthonectid species were assembled and annotated previously [11,25]. They have extremely low GC content—about 17–21%, which is very close to the lowest for Metazoa, with the record lowest content of 13% reported for a parasitic wasp *Diadegma semiclausum* (Hellén, 1949) [26]. Here, we re-assembled and re-annotated the mitochondrial genome of orthonectid *Intoshia variabili* (Alexandrov & Sljusarev, 1992) [11]. Also, we completed the mitochondrial genome of *Intoshia linei* Giard, 1877 via Sanger sequencing of missing regions. In these updated versions of mitochondrial genomes, we found the previously missing *atp8*, *trnQ*, and *trnR* genes in *I. linei* and *cox3*, *nad2*, and *atp8* genes in *I. variabili* (Figure 1, File S1). The presence of the *atp8* gene distinguishes the orthonectids from parasitic flatworms Neodermata [27], which were repeatedly discussed as probable kindred of the orthonectids [28,29]. Large portions of the mitochondrial genomes in *Intoshia* species can be co-aligned. Their genomes differ in the position of the *cox2-rrnS* region, and the position and length of the non-coding region, situated between *nad4* and *nad5* genes in *I. linei*, and *nad2* and *nad5* genes in *I. variabili*. The size of the non-coding region is unusually large in *I. variabili*, reaching 5606 bp according to the assembly produced with NOVOPlasty [30].

RNA-seq assembly [6] revealed four primary transcripts in *I. linei*: three polycistronic and one monocistronic containing only 12S rRNA. There are two bi-directional transcription start points in the mitogenome, which represent potential control regions (*CRs*) [31]: between *trnF* and *nad1* (*CR1*) and between *rrnL* and *trnM* (*CR2*). *CR2* is the transcription origin for both rRNA genes. There are two convergence points for the transcription: between *trnK* and *nad5* and between *rrnL* and *trnC*. The first convergence point coincides with an inverted repeat region, which terminates transcription on both sides. The inverted repeat is 472 bp long, which exceeds the length of the illumina reads. The primary structure of this region was clarified using PCR and Sanger sequencing. In the RNA library, the transcripts of seven protein-coding genes are polyadenylated at their 3′-ends (Figure 1). We did not observe polyadenylated tRNAs or rRNAs.

### 2.2. Bayesian Approach to Orthonectid Phylogeny

**Figure 2:**
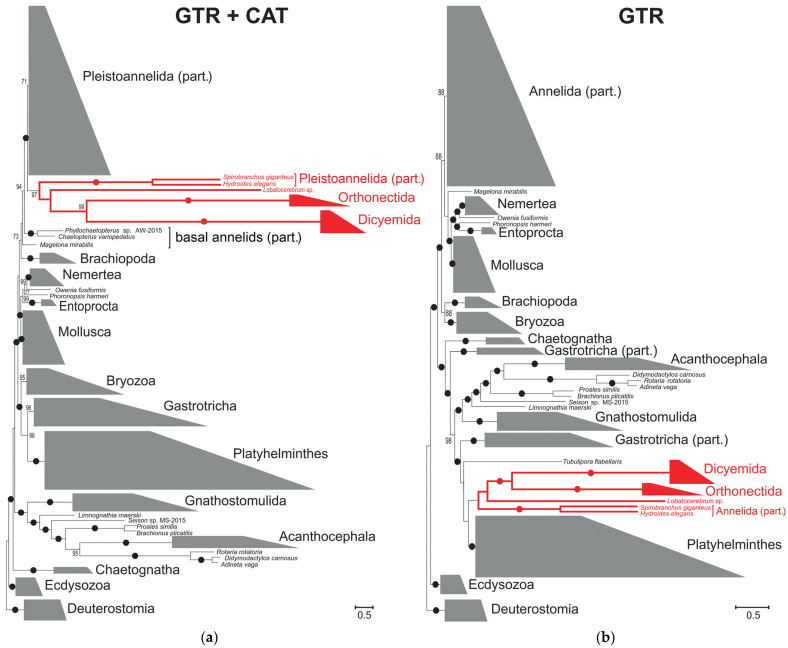
Bayesian trees reconstructed with the concatenated dataset of 12 mitochondrial proteins (**a**) under the GTR+CAT+Γ and (**b**) GTR+Γ models. Numbers at the branches indicate Bayesian posterior probabilities (in %), values below 70 are not shown, values of 100 are marked with dots. Long-branched annelids, orthonectids, and dicyemids are marked in red. For taxonomic sampling details, see Figure S1 and Table S1.

To examine the spiralian phylogeny and determine the position of orthonectids among different groups of Lophotrochozoa, Bayesian trees were constructed with a concatenate of 12 mitochondrial proteins using the site-heterogeneous GTR+CAT+*Γ* and site-homogeneous GTR+*Γ* models (Figure 2). In the GTR+CAT+*Γ* tree, most annelids form a monophyletic group, with the exception of the earliest lineages of modern annelids, as was previously reported for mitochondrial phylogenies [32]. All long-branched annelids (*Lobatocerebrum* sp., *Spirobranchus giganteus* (Pallas, 1766) and *Hydroides elegans* (Haswell, 1883) together with orthonectids and dicyemids form a cluster with a posterior probability of 0.99 before annelid crown radiation (Figure 2a). In the tree reconstructed using the site-homogeneous model GTR+*Γ* (Figure 2b), the annelid long-branched cluster including orthonectids and dicyemids groups with other long-branch lophotrochozoans outside Annelida. The clustering of divergent sequences is characteristic of long-branch attraction artifact, and in this case, violates the established phylogenetic relationships [33,34,35,36,37]. In both trees, however, Orthonectida groups with Dicyemida with posterior probabilities of 0.97 or 1.0, and with the annelid *Lobatocerebrum* sp. as the closest relative.

Taxa may be mistakenly grouped in phylogenetic analyses as a result of nucleotide or amino acid composition biases, inadequacy of applied models of molecular evolution, homoplasies arising at rapidly evolving sites. These erroneous groupings often manifest themselves as clusters of long-branched species in the phylogenetic tree [15,16]. To examine the contradictory placement of these long-branched species, we decided to use an alternative method for determining kinship that does not rely explicitly on the assumptions of substitution models and involves only rare synapomorphies, suppressing all signals from more variable, homoplasy-prone characters.

### 2.3. Synapomorphies of Annelida and Orthonectida

Mitochondrial gene orders in orthonectids are not identical to each other and also differ greatly from other Bilateria. However, orthonectids share the joint gene pair *cytb-trnW* with the majority of annelids [32,35,37]. The tRNA gene arrangement is less conserved than that of mitochondrial protein-coding genes (PCGs) [35,38], so it is unlikely that *trnW* retained its position when the order of PCGs underwent global reorganization. In other words, we reject the possibility that the *cytb*-*trnW* pair was inherited by orthonectids and annelids from a common ancestor of Lophotrochozoa, but has been lost in most lineages. However, the probability that the *cytb*-*trnW* pair arose independently in annelids and orthonectids needs to be assessed. In RefSeq, among all Metazoa except annelids, this character is present in only six species out of 15,885 (0.04%), four of them are mollusks of the genus *Panopea*, two are gnathostomulids. Obviously, in these taxa, *cytb*-*trnW* arose independently of annelids. Thus, the *cytb*-*trnW* is characterized by a low level of independent occurrence (homoplasy) of 0.04%. This value may be overestimated: if we assume the inheritance of the feature by *Panopea* and gnathostomulids from their ancestors, then the feature is recorded only twice outside of annelids and orthonectids. Also, it is possible to give an upper estimate: if the orthonectid gene order is randomized, then *trnW* gene can follow any other gene or CR in a certain orientation with a probability of ½ × ^1^/_37_ ≈ 1.35%. Among annelids, the *cytb*-*trnW* is common in pleistoannelids and *Magelona mirabilis* (Johnston, 1865), but not in other basal clades (*Owenia fusiformis* Delle Chiaje, 1844, Chaetopteridae, Sipuncula and Amphinomidae). The *cytb*-*trnW* feature was either preserved during the independent evolution of the oldest annelid lineages or arose after the separation of the basal clades and independently in *M. mirabilis*. The current lack of information about the mitogenomes of basal branched annelids leads to difficulties in clarifying the history of annelid gene rearrangements.

The gene order rearrangement analysis is inapplicable for dicyemids, since most of their mitochondrial genes are localized on individual replicons [9,39].

The crown annelid species—pleistoannelids—share numerous apomorphic features in the mitochondrial gene order [32,35,40,41]. For example, *Owenia fusiformis* and *Chaetopterus variopedatus* Renier, 1804, representatives of early lineages of annelids, retain the plesiomorphic gene pair *nad1*–*nad6* (and some tRNA genes between them), whereas in pleistoannelids, amphinomids, and sipunculids *nad6* is a part of the conserved block *cox3-nad6-cytb* (ignoring the tRNA genes). The plesiomorphy of *nad1*–*nad6* in orthonectids argues for their branching from the common annelid tree before the divergence of pleistoannelids and amphinomids/sipunculids [8,25].

A similar method for the detection of individual synapomorphies in annotated proteins was applied to other groups of Lophotrochozoa that were previously considered potential relatives of orthonectids (Annelida, Mollusca, Platyhelminthes and Gastrotricha). In the first step, orthonectids and dicyemids were excluded from the alignment, and then candidate synapomorphies of the listed taxa were found using the maximum likelihood method (see details in the Methods Section). In the second step, candidate sites were screened against RefSeq and for similarities with orthonectids and dicyemids.

Nine synapomorphies with homoplasy levels below the threshold of 3% were found for Annelida (Table 1).

Orthonectids *I. linei* and *I. variabili* have four annelid synapomorphies, while *Rhopalura litoralis* shares only two of them, in cox1 and cytb (Table 2). Dicyemids, on the other hand, have no annelid synapomorphies.

A range of 83% to 99% of annelids share common amino acids with orthonectids at these four sites, while in other Metazoa it ranges from 0.1% to 2.7%, which can be interpreted as low levels of homoplasy. Interestingly, each of the synapomorphies is localized in a separate polypeptide (NAD6, Cytb, COX1, and ATP6) belonging to one of four spatially separated multisubunit protein complexes (electron transport chain complexes I, III, IV, and ATP synthase, respectively).

**Figure 3:**
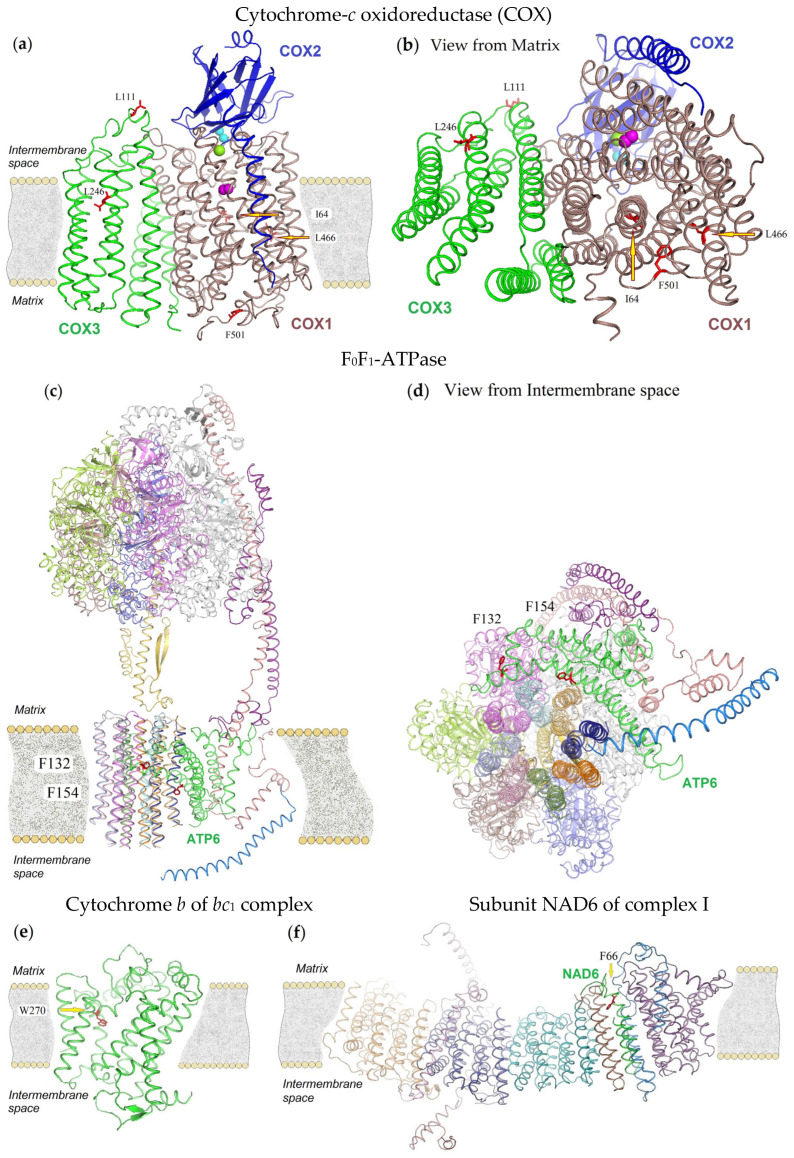
Locations of synapomorphies in mitochondrially encoded subunits of *I. linei* mitochondrial complex IV (COX—cytochrome-*c* oxidoreductase), complex V (F_0_F_1_-ATPase), complex III (CoQ:cytochrome-*c* oxidoreductase, i.e., *bc*_1_ complex) and complex I (NADH:CoQ oxidoreductase). 3D-structures of mitochondrial complexes were predicted on the basis of homology modeling. Synapomorphic residues in subunits are shown in sticks (red), in some places marked with yellow arrows and denoted in one-letter code. (**a**,**b**) COX core subunits: COX1 (brown), COX2 (blue) and COX3 (green). Spheres depict Cu_A_ redox center in COX2 (cyan), Mg ion in COX1 (green), hydrogen peroxide in COX1 active center (magenta). (**c**,**d**) Membrane subunits (F_0_) of F_0_F_1_-ATPase and subunits contacting them are shown in color, subunit ATP6 among them is green-colored, other peripheral subunits (F_1_) are colored in gray, purple, and olive and can be seen as (**c**) a mushroom cap protruding from the membrane. (**e**) Intrinsic membrane protein, mitochondrially encoded cytochrome *b*, which is the subunit of complex III. (**f**) Intrinsic membrane subunits of complex I, mitochondrially encoded subunit NAD6 among them is green-colored; other subunits are not shown. (**a**,**c**,**e**,**f**) View of protein subunits in a slice of the mitochondrial membrane, the slice runs perpendicular to the membrane surface and is directed from the mitochondrial matrix (Matrix) to the intermembrane mitochondrial space (Intermembrane space); (**b**) view from the Matrix; (**d**) view from the Intermembrane space.

The detected annelid synapomorphies in the COX core subunits, COX1 and COX3, are spatially distant from each other. The distance between these residues within individual subunits ranges from 16 to 31 Å in COX1 and 27–64 Å in COX3, and from 16 to 59 Å between residues located in different polypeptides (Figure 3a,b). Of the three synapomorphic residues of the catalytic subunit COX1, two residues, I64 and L466, are located in the 2nd and 12th α-helices, respectively, in the center of the lipid bilayer at a distance of 16 Å from each other. Another residue, F501, belongs to the loop exposed in the matrix and is located at a distance of 28 and 31 Å from I64 and L466, correspondingly. These three residues of the COX1 subunit are also significantly distant from the pocket with the catalytic center. This suggests the absence of both direct interactions between these residues and interactions resulting from common function. Also, their involvement in the formation of subunit contacts in the cytochrome oxidase enzymatic complex is not evident. Additionally, COX core subunits 1 and 3 are highly hydrophobic membrane proteins that typically span the inner mitochondrial membrane 12 and 7 times, respectively [42]. Consequently, such mitochondria-encoded proteins must undergo multiple transitions from the matrix to the inner mitochondrial membrane and back during synthesis and then assemble into a functional enzyme complex. At present, the process of COX maturation and assembly is poorly understood. It is known that in *Saccharomyces cerevisiae* about three dozen different helper proteins are involved in the regulation of COX transcription, translation and processing [43]. Among them, co-translational membrane insertion helper proteins are known; the participation of chaperonins, without which assembly cannot be envisioned, is also suggested. As we see, synapomorphies in mitochodria-encoded subunits in representatives of Annelida (Table 1 and Figure 3a–f) are represented mainly by hydrophobic amino acid residues, which may be necessary to participants in the complex assembly process of enzyme complexes. The possible reason for the observed taxon-specific conservation of these residues is not a functionally determined co-evolution, but rather their involvement in binding to chaperone proteins, which is crucial for the stabilization, folding, and assembly of the growing polypeptide chains in the inner mitochondrial membrane. Therefore, we assume that mitochondrial protein synapomorphies in annelids arose independently of each other and that their emergence represents unrelated evolutionary events.

We calculated the probability of independent occurrence of four or more of these features using the frequency values of nine features and taking into account the conditional probabilities for three pairs of dependent features (4 + 9, 6 + 7 and 6 + 9, Table S2). The resulting value was 4.2 × 10^−8^ (Table 3). The NCBI RefSeq database (15,885 Metazoa species at the time of analysis) has no species outside of annelids with four or more annelid synapomorphies (according to the accepted thresholds). Taking into account the independent rearrangement of gene order (the *cytb*-*trnW* pair) with a homoplasy value of 0.04%, the final probability of evolutionarily independent occurrence of synapomorphies in orthonectids and annelids is 1.64 × 10^−11^. If we take the 2% threshold, then annelids are left with six synapomorphies, of which orthonectids have three synapomorphies. In this case, the probability of independent occurrence of three or more of these features is 0.05. If we take the 1% threshold, then annelids are left with three synapomorphies, of which orthonectids have two synapomorphies. In this case, the probability of independent occurrence of two or more of these features is 0.014.

Orthonectids lack five annelid synapomorphies. In three cases, orthonectids share the same amino acid as 4% or less of non-annelid Metazoa, which are distributed throughout the phylogenetic tree and do not cluster into a monophyletic taxon. In two cases they share the same amino acid as 96% (feature 7) and 19% (feature 3) of non-annelid Metazoa. Orthonectids share symplesiomorphic character for feature 7 not only with the outgroup to annelids, but also with basal annelid branches (*Owenia fusiformis* and *Magelona mirabilis*). Feature 3 is not very stable in annelids (Table 1) or other Metazoa, where various hydrophobic amino acids (L, I, V, M) are often found at this site.

After removing the four columns containing synapomorphies of orthonectids and annelids from the alignment, orthonectids no longer group with other annelids in the phylogenetic analysis with the site-heterogeneous GTR+CAT+*Γ* model (Figure S2). The reconstructed tree also undergoes other changes: *Spirobranchus giganteus* and *Hydroides elegans* no longer group with other long-branched annelids, *Lobatocerebrum* sp. localizes outside annelids, and posterior probabilities within Annelida decrease. Therefore, the removed alignment sites make a significant contribution to the phylogenetic signal. The posterior probability of the orthonectid and dicyemid grouping does not decrease.

Due to the fragmented assembly of *Lobatocerebrum* sp., there are no data for this species on the states of features no. 2, 4, 8, and 9. Coincidentally, these are the features that annelids and orthonectids have in common. Among the other five features, *Lobatocerebrum* sp. has one annelid synapomorphy (no. 6) in COX3. Thereby, *Lobatocerebrum* sp. has lost at least four annelid synapomorphies. Since Lobatocerebrida belongs to Annelida [33] we can see it as another example of feature loss due to high evolutionary rates.

No synapomorphies characterized by a low level of homoplasy in mitochondrial proteins of dicyemids and any other of the studied groups of Lophotrochozoa were found.

### 2.4. Placement of Orthonectida in Annelida

**Figure 4:**
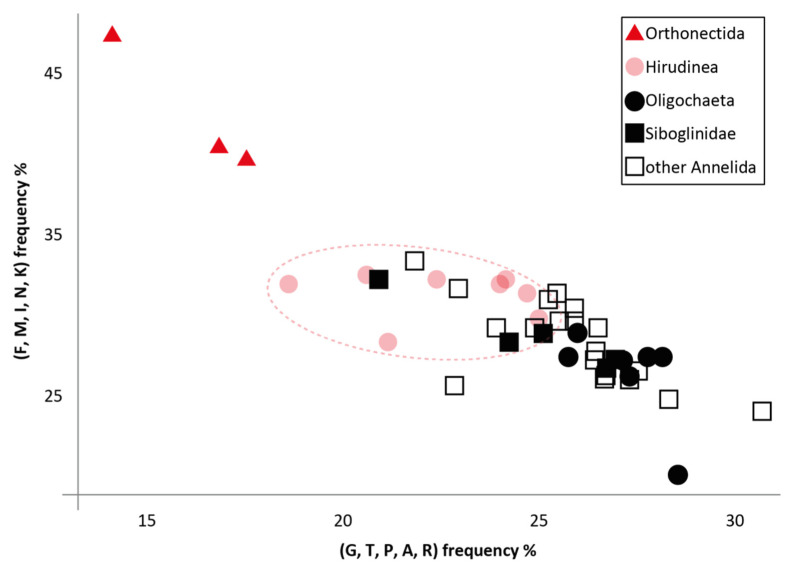
Scatterplot between the sums of the occurrences (in percentage) of amino acids with AT-rich codons (F, M, I, N, K) and amino acids with GC-rich codons (G, T, P, A, R) in proteins encoded by mitochondria of annelids.

The exact position of orthonectids within annelids remains an unresolved issue. Previously, the relationship of orthonectids with leeches (Hirudinea) was proposed based on mitochondrial gene sequences [11]. We found that leeches have reduced GC% in their mitogenomes, which affects the amino acid composition (Figure 4). Although in the case of leeches, this deviation is not as large as in orthonectids, we hypothesize that it may influence tree reconstruction.

We examined various taxonomic groups of annelids (Clitellata, Errantia, Hirudinea, Oligochaeta, Pleistoannelida, Sedentaria, Siboglinidae and Syllidae) for potential synapomorphies with orthonectids. Putative synapomorphies, selected for the homoplasy levels and stability within the group, were discovered in three pleistoannelid groups—Clitellata, Hirudinea, and Siboglinidae (Table 4).

In all four cases, orthonectids possess only a small portion of the group’s synapomorphies. Therefore, we considered a hypothesis that orthonectids lost most of the synapomorphies derived from the common ancestor of the group due to the high rate of molecular evolution. Thus, in addition to calculating the probabilities of independent (i.e., due to homoplasy) emergence of synapomorphies, the probabilities of loss of synapomorphies by orthonectids if they actually belong to this group were also calculated (Table 5).

Despite the comparable number (three or four) of synapomorphies of orthonectids for the four studied annelid groups, the probability of their independent emergence in the case of annelids is 10 to 100 times lower compared to the three groups of pleistoannelids. The main contribution to this difference is the difference in the levels of homoplasy for these synapomorphies—for annelids it averages to 1.5% (Table 1), and for other groups it varies from 1.85% to 2.33%. Since at the time of analysis there were 157 annelids in the RefSeq, we expect to find an average of 0.2, 2.2, and 2.7 annelids that independently acquired as many or more synapomorphies as orthonectids due to homoplasy. Therefore, the similarity of orthonectids with Hirudinea and Siboglinidae may be due to homoplasy. At the same time, the probability of the orthonectids losing five of the nine synapomorphies of annelids is 33%, which does not contradict the hypothesis that the remaining four characters were inherited from a common ancestor. In the case of Clitellata synapomorphies, the probability of orthonectids losing 9 out of 12 characters is 1.5%. The probability of orthonectids losing synapomorphies of the Hirudinea and Siboglinidae groups is much lower—2.3 × 10^−6^ and 3.3 × 10^−11^ respectively. Based on these calculated probabilities, the hypothesis that orthonectids belong to leeches is highly unlikely. However, the closer relationship of orthonectids with Clitellata and the grouping of orthonectids with leeches within Clitellata cannot be excluded.

### 2.5. Synapomorphies of Orthonectida and Dicyemida

The grouping of orthonectids and dicyemids in phylogenetic trees constructed using different models if not artefactual might indicate the presence of shared conserved characters. Potential synapomorphies and homoplasies of Mesozoa were searched using the initial alignment and a fixed topology from Figure 2a (see details in Materials and Methods Section). In total, we found 31 mesozoan synapomorphies that are simultaneously stable in both groups and rare in other Metazoa (occurring in less than 3% of metazoan species in RefSeq) (Table 6):

In view of the high rate of evolution of both orthonectids and dicyemids, the detection of even a large number of potential synapomorphies in these groups might simply be a corollary of a higher incidence of homoplasies. To test this hypothesis, we examined the occurrence of traits falling under the synapomorphies condition (low homoplasy and high within-group stability) for three groups of Lophotrochozoa with long branches—Orthonectida, Dicyemida, and Neodermata—the crown group of flatworms. Some observations indicate the existence of taxon-specific patterns of amino acid substitutions in mitochondrial proteins, presumably due to epistatic interactions between sites [44]; therefore, we decided to use data not for the whole RefSeq, but only for lophotrochozoans—1294 species at the time of the study. Since no synapomorphies with the basal group of flatworms have been found in both orthonectids and dicyemids, potential synapomorphies of neodermatids and orthonectids/dicyemids would be convergently acquired traits. Thus, in this analysis, neodermatids will act as a “negative control” on the detection of synapomorphies between lophotrochozoan groups with high evolutionary rates. The expected number of common features was calculated using the following approximate procedure. For each of the three groups, all positions of the mitochondrial protein alignments were found such that the frequency of a particular amino acid residue within the group exceeded the 70% threshold, while among other Lophotrochozoa outside of these three groups, the frequency of this residue would not exceed 3%. For each such position, the frequency of the corresponding residue among other Lophotrochozoa was calculated. The expected number of rare features in common with a given group in a randomly selected representative of other Lophotrochozoa can be estimated as the sum of frequencies across all such positions. The expected number of common features shared with at least one of the two groups was estimated as the sum of expected values for each of these groups. As a result of the calculations, the following results were obtained (Table 7):

In all cases, the observed number of potential synapomorphies exceeds the number of expected ones. In the cases of Orthonectida+Neodermata and Dicyemida+Neodermata, acting as a “negative control”, observed/expected ratios coincides, and in the case of Orthonectida+Dicyemida, the observed/expected ratio exceeds the “negative control” by a quarter. We hypothesize that these calculations indicate that the grouping of orthonectids and dicyemids is not random—at least one in five potential mesozoan features (i.e., 4.5 out of 22) is a true synapomorphy.

If orthonectids and dicyemids are the closest (sister) taxa, dicyemids are a group of annelids, a group so far removed from the typical representatives that traces of relatedness have been lost at both morphological and molecular levels at least within mtDNA. Since the dicyemids do not have any of the nine synapomorphies of annelids, we calculated the probability of the dicyemids losing the nine synapomorphies of annelids (if they belong to Annelida), which is 5%. In this case, it is possible to recover the placement dicyemids in annelids only due to the presence of a related divergent group of orthonectids. However, additional methods of analysis and large volumes of data are needed to make a final judgment on the affiliation of dicyemids to a specific group of lophotrochozoa or to find their closest sister taxon.

### 2.6. Orthonectida and Lobatocerebrida

In the phylogenetic trees, the closest relative to orthonectids and dicyemids is an annelid *Lobatocerebrum*—free-living meiobenthic worm with an unusual body plan. The inclusion of *Lobatocerebrum* in annelids was previously shown based on transcriptomic data [33]. The close relationship of the orthonectids and lobatocerebrid raises the question about specific similarities of these taxa in the mitochondrial gene order, and other molecular and morphological characters. We assembled the raw transcriptome reads of *Lobatocerebrum* sp. from the Sequence Read Archive of NCBI and found transcripts of 13 PCGs (File S1). Five contigs contained tRNA sequences (*cox2*-*atp8*-*cytb*, *nad4L*-*nad4*-*trnK*, *trnW*-*atp6*, *trnL2*-*nad1*, and *nad2*-*trnD*-*trnY*-*nad3*). Four PCGs that are located at the 3ʹ-ends of the assembled contigs (*atp6*, *cox1*, *cytb*, and *nad3*) and the *nad2* gene, which is included inside of a longer contig, were observed to have reads terminating with poly(A) stretches downstream of the TAA stop codons. Two genes—*cox1* and *cytb*—produce poly(A) stretches in transcripts from both *Lobatocerebrum* sp. and *I. linei*; *atp6*, *nad2*, and *nad3*—only in *Lobatocerebrum* sp.; *atp8*, *cox3*, *nad1*, *nad4*, and *nad4l*—only in *I. linei*. A combination of unique and plesiomorphic gene adjacencies is seen in the partial mitogenome of *Lobatocerebrum* sp. assembled from the transcriptomic reads. The unique adjacencies are not surprising given the rate of substitutions in the mitochondrial sequences of *Lobatocerebrum* sp. Among plesiomorphic traits, the *nad4L-nad4* and *trnL2-nad1* adjacencies are very conservative and widespread among Bilateria [45,46], whereas the adjacency of *trnW-atp6* is shared only by crown annelids and species with long branches (orthonectids, *Spirobranchus giganteus* and *Hydroides elegans*) or high rearrangement rate. The partial data on the mitochondrial gene order present evidence of the kinship of *Lobatocerebrum* sp. with the annelids but do not lend support for its close relationship to the orthonectids.

Since both groups, Orthonectida and Lobatocerebrida, have long branches, we examined the possible influence of nucleotide composition bias on their grouping. Using RefSeq data, we calculated average nucleotide composition values in mitochondrial proteins of all annelids excluding long-branched species: orthonectids, *Lobatocerebrum* sp., *Spirobranchus giganteus*, and *Hydroides elegans*. Both orthonectids and *Lobatocerebrum* sp. strongly differ from the average 34% GC seen in Annelida: *I. linei* and *I. variabili* have 17% GC, *Rhopalura litoralis*—21% GC, and the partial mitochondrial genome of *Lobatocerebrum* sp. has 23% GC. Nucleotide bias is also reflected in the amino acid bias due to the preponderance of AT-rich codons. Changes in amino acid composition, in turn, can lead to artefactual groupings in phylogenetic trees. However, removal of the AT-rich codon amino acids (F, M, I, N, K) from orthonectid and lobatocerebrid sequences did not affect their grouping in the reconstructed phylogenies (Figures S3–S5). Therefore, the grouping of Lobatocerebrida with Mesozoa cannot simply be explained as a consequence of similar composition biases.

Additionally, we searched for synapomorphies of *Lobatocerebrum* and Mesozoa using the approach described above. As a result, 10 synapomorphies common to *Lobatocerebrum* and at least one of the Mesozoan groups, orthonectids and dicyemids, were found (Table 8).

Interestingly, Orthonectida and Lobatocerebrida share some similarities in their morphology. The worm-like stage of orthonectids is characterized by an unusual arrangement of the longitudinal muscles that lie outside the circular muscles [3]. According to the original description [47,48], *Lobatocerebrum* is another taxon with the same unusual muscle arrangement, which differs from the arrangement found in most annelids [49,50]. The muscle arrangement could be considered a probable synapomorphy of the orthonectids and Lobatocerebrida; although, according to a recent observation [51], the transverse muscular ring complexes in *Lobatocerebrum* have been misidentified as internal circular musculature. Therefore, a more detailed study of the body wall of the orthonectids is necessary to decide if they share the muscular arrangement with *Lobatocerebrum*. Only smooth muscles were detected in *Lobatocerebrum* sp. [51]; troponin, an obligatory component of the bilaterian striated muscles, was not detected in the genome of *I. linei* [6]. However, we found troponin mRNA in the nuclear transcriptome of *Lobatocerebrum* sp., which might indicate either independent reduction in striated muscles or progressive stages in the reduction-initial in lobatocererbrids and final in orthonectids (Figure S6).

The nervous systems of orthonectids and lobatocerebrids are similar in that neurons are concentrated in one dorsal ganglion. The brain is evidently lobular in lobatocerebrids and bilaterally symmetric in orthonectids, at least in the arrangement of the serotonergic neurons in three pairs, which are the only neurons accurately identified thus far [3]. The brain in orthonectids lies at some distance from the anterior end of the body and sends three processes forward, which are not analogous to any other spiralians except the rostral nerves of the *Lobatocerebrum* [51], and two lateral processes back, which are located as the widely spaced ventrolateral nerve cords of the *Lobatocerebrum*. The longitudinal nerves of most annelids are arranged differently, as a pair of closely spaced cords that carry serial ganglia linked by transverse commissures and run through the ventral part of the body. The organization of the longitudinal nerve cords of the orthonectids and lobatocerdrids is similar but not specific to the two taxa and is also found in some archiannelids, such as Protodrilidae, Dinophilidae, and Nerillidae. We can conclude that the morphological features do not readily support the close relationship of orthonectids and lobatocerebrids, but also do not contradict it. As a result, the available morphological data do not give decisive arguments for establishing the position of orthonectids.

### 2.7. Morphological Similarities Between Orthonectida and Annelida

The fact that the “phylum” Orthonectida forms one of the branches of Annelida in a phylogenetic tree was somewhat anticipated by the morphological analyses and in view of their nature as members of the Lophotrochozoa [34,52]. Despite the extremely simplified morphology, orthonectids have an annelid type of microvillar cuticle [53,54] and metameric circular muscle fibers [3,55,56] in addition to the external metamerism from the ciliary rings of the epithelium. Microvillar cuticle and metamerism are not specific to annelids but rather to most lophotrochozan phyla [57,58,59] with the exceptions of Nemertea, Platyhelminthes, Gastrotricha, Gnathifera, and Dicyemida. Considering the evidence of dissepiments in adult brachiopods and phoronids [60], metamerism can be a plesiomorphic state of Lophotrochozoa, hence its retention by the orthonectids cannot serve as a particularly convincing argument in favor of their origin from annelids. Annelids display some other cases of extreme simplification of morphology, for example, in dwarf males of Echiura [61], Dinophilidae [62], Siboglinidae [63], and Spionidae [64]. In particular, males of *Dinophilus gyrociliatus* are similar to adult orthonectids in body size and number of cells. The dioeciousness of microscopic mature stages indicates that orthonectids originated from larger animals, rather than from a small turbellaria-like ancestor, for which a small body size has a clear correlation with hermaphroditism. The life cycle of orthonectids shows only superficial similarity to that of parasitic flatworms, such as flukes [65]. Germinal cells in orthonectid plasmodium generate mature individuals, and not parthenites, unlike rediae. Finally, the orthonectid spermatozoa with one flagellum preserve the plesiomorphic structure and not shared apomorphies with the flatworms [66]. A set of morphological plesiomorphies (sperm structure, gonochory, epithelial and muscular metamery of mature stages) sets apart orthonectids from the groups comprising Platyhelminthes, but plesiomorphies cannot in principle exclude their sister group relationship.

### 2.8. In Lieu of a Conclusion: Prospects for Optimizing

The current breadth of the reference sequence database (RefSeq, NCBI) allows application of nonstandard methods for inferences of phylogenetic signal. However, the implementation of the synapomorphy detection algorithm utilized in the present study provides only coarse assessment of probabilities, as it does not account for the tree structure of species and character evolution, and is skewed by the highly unbalanced representation of taxa in the database. Nucleotide sequences evolve at different rates across taxa, and amino acid sequences in orthologous proteins may have different substitution patterns [44]. The RefSeq database is enriched in vertebrate and arthropod species, which causes bias in calculation of statistical parameters for the entire database and arouses suspicion in good applicability of this calculation. If we will try to overcome this bias by use only lophotrochozoan species, some taxa (Mollusca and Neodermata) will still be overrepresented, revealing another bias. If we exclude Mollusca and Neodermata, the remaining taxa are still unevenly represented, etc. In a sense, the natural sampling is also uneven, due to the evolutionary success and species radiation of a few lineages. The way big data (i.e., the entire RefSeq database) is used here can presumably be improved by clever sampling or weighting the contributions of each species or taxa, which would still boost the accuracy without resorting to costly inferences of phylogeny and the evolution of characters on a tree. However, such approaches require careful design and depend on a reliable topology in deep nodes such as phyla.

Moreover, justifying the selection of synapomorphy intra-group stability and homoplasy thresholds will also clarify the identification of significant rare evolutionary events. However, the very concept of “rare” is relative and not strictly defined, which complicates the selection of a universal algorithm for finding such events.

Multiple lines of evidence now point to the close relationship of Orthonectida and Annelida, yet their exact placement within annelids or the putative close relationship with Lobatocerebridae remain unresolved. The exact placement of Dicyemida in Lophotrochozoa is also questionable. Data from the analysis of mitochondrial gene phylogeny and synamorphies, and some of the previous phylogenomic studies, argue in favor of a group of orthonectids and dicyemids, the classical Mesozoa, although the phylogenetic signal in mtDNA favoring the group is weak and cannot be conclusively disentangled from arteficial long branch attraction. Improved sampling of these enigmatic animals may yet provide better grounds for the application of various phylogenetic methods to resolve their position among Lophotrochozoa.

## 3. Materials and Methods

### 3.1. Mitochondrial Genome Assembly and Annotation

Mitochondrial genome of *Intoshia variabili* was re-assembled (File S1) with NOVOPlasty [30]. The mitogenome sequences were annotated with the MITOS2 [67] using the genetic code for invertebrate mitochondria. Transfer RNA genes were detected using the MiTFi program [68]. The annotations were refined manually using alignments of protein-coding sequences and the GenomeView browser [69]. Mitochondrial genome maps were constructed using the OGDRAW version 1.3.1 software [70].

### 3.2. Determination of the Primary Structure of Non-Coding Region in the Mitogenome of I. linei

Orientation of the central region of the long inverted repeat, forming the non-coding region (1145 bp in length) in the mitogenome of *I. linei*, was determined by polymerase chain reaction (PCR) using primers rhair (5′-CCCAAAACACCTATTTCTGCTGGC-3′), d14390 (5′-TATATGTATTTCATAGAAGGAGG-3′) and r480 (5′-GAGATTTTTAAGCTCAAGAAGAGTACC-3′) specially designed for this study in Lumiprobe RUS Ltd. (Moscow, Russia). Due to suppression of amplification by the long-inverted repeat, the PCR product was obtained only after treatment of native *I. linei* DNA with restriction enzyme EcoP15I, the site for which (CAGCAG) was predicted in the central region of the inverted repeat using the preliminary assembly. The PCR cycling conditions included denaturation at 95 °C for 5 min, followed by 40 cycles of denaturation at 95 °C for 30 s, annealing at 55 °C for 30 s, extension at 72 °C for 3 min, and final extension at 72 °C for 5 min. PCR products were agarose gel-purified with Cleanup Mini kit (Evrogen, Russia). Purified amplicons were sequenced with an Applied Biosystems 3730 DNA Analyzer (Thermo Fisher Scientific, Waltham, MA, USA).

### 3.3. 3D-Modeling of Mitochondrial Proteins

The structure homology modeling of the predicted subunits of respiratory chain complexes I, III, IV and V, i.e., NADH:CoQ oxidoreductase, *bc*_1_-complex, cytochrome-*c* oxidoreductase (COX) and ATPase, was achieved via the SWISS-MODEL Server and the SWISS-MODEL Workspace/GMQE homology modeling software [71] using the crystal structure of *Bos taurus* COX in the fully oxidized state ([72], PDB: 3ABM.1, 44.79% identity, 1.95 Å resolution), *B. taurus* F_0_F_1_-ATPase ([73], PDB: 7AJF.1, 56.27% identity, 9.2 Å resolution), *B. taurus bc*_1_ in complex with 2-pyrazolyl quinolone inhibitor WDH2G7 ([74], PDB: 6HAW.1, 36% identity, 3.45 Å resolution), mouse mitochondrial complex I in the active state ([75], PDB: 6g2j.1, 25.68% identity, 3.3 Å resolution) as templates. Protein structures were visualized using the PyMOL Molecular Graphics System, Version 1.2r1 Schrödinger, LLC. Prediction of mitochondrial presequences, cleavage sites and specific sequence motifs was performed using MitoFates Webserver [76].

### 3.4. Phylogenetic Analysis

The protein-coding gene sequences of orthonectids were aligned with sequences from 55 lophotrochozoans which were selected to cover all Lophotrochozoan phyla with available data. Additionally, we utilized transcriptomic data from 12 species to sample poorly represented taxa (Supplementary: Table S1). All predicted amino acid sequences were aligned with MAFFT online [77]. The highly variable ATP8 protein has been omitted. The PhyloBayes tree inferences were performed in PhyloBayes MPI 1.7b [78] under GTR+CAT+*Γ*4 and GTR+*Γ*4 models. Models were selected based on previous research, which revealed that using the CAT model implemented in PhyloBayes is important for alleviating the issues with phylogenetic reconstructions [9]. The resulting Bayesian trees were visualized in MEGA 11 [79].

### 3.5. Synapomorphy Search

**Figure 5:**
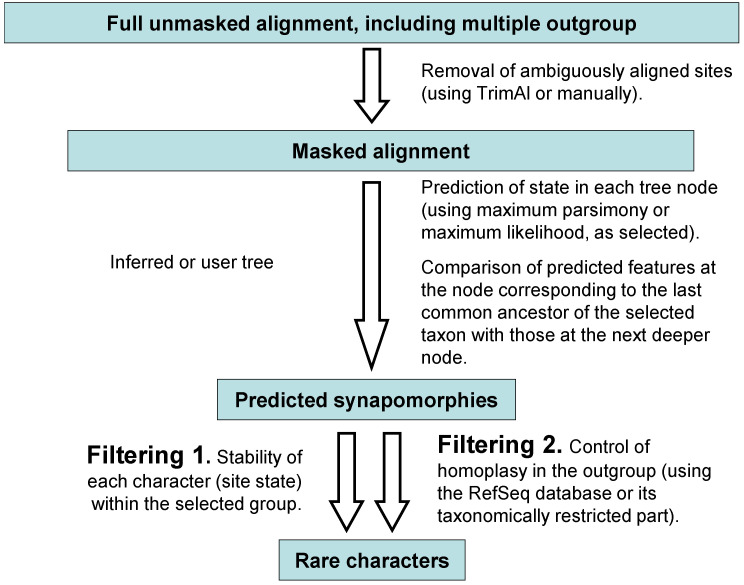
An algorithm for finding characters with low levels of homoplasy in amino acid or nucleotide alignments.

The search for characters with a low level of homoplasy was carried out in several steps (Figure 5), including the search for synapomorphies and their subsequent filtering by two parameters.

Search for synapomorphies was performed using ancestral state reconstruction in IQ-TREE [80] and the GTR+CAT+*Γ*4 model Bayesian phylogenetic tree (Figure 2a). After calculating the ancestral states for each node of a given tree, we attributed apomorphic characters for each lophotrochozoan taxa proposed in various studies as relatives of orthonectids: Annelida, Platyhelminthes, Mollusca and Gastrotricha (Chaetonotida). A character was considered apomorphic if it was reconstructed as ancestral for a taxon but not for the next deeper node on the tree. Next, we examined three orthonectid species (*I. linei*, *I. variabili*, and *Rhopalura litoralis*) for the presence of selected features. If a feature was present in at least two orthonectids, it was noted as a potential synapomorphy of this group of Lophotrochozoa and orthonectids. Next, we calculated the occurrence of this feature outside the original group among all Metazoa from the RefSeq database (in some cases we used the taxonomically restricted group Lophotrochozoa instead of all Metazoa). There were a total of 15,585 Metazoan mitochondrial genomes in RefSeq at the time of analysis on March 2025 selected with filters “RefSeq” and “Mitochondrion” and search parameter “Metazoa”. Features found in more than 3% of Metazoa outside the group (i.e., occurring as homoplasy with a frequency of more than 3%) were not considered. The remaining features were examined for resistance to evolutionary changes within the group—characters that were lost in more than 40% of the group’s representatives were not considered. The search for synapomorphies of orthonectids and different annelid groups was performed similarly, although different alignments and trees were obtained—there were 96 annelids and four representatives of Mollusca as an outgroup. The following partially included in one another annelid groups were tested: Clitellata, Errantia, Hirudinea, Oligochaeta, Pleistoannelida, Sedentaria, Siboglinidae and Syllidae.

To find common phylogenetic signals in the mitochondrial PCGs of orthonectids and other Lophotrochozoan taxa (Annelida, Mollusca, Platyhelminthes and Gastrotricha), synapomorphies were searched for these taxa. We did not find any potential synapomorphy with a homoplasy level of less than 3% shared by orthonectids and Mollusca, Platyhelminthes and Gastrotricha, which were previously clustered with the orthonectids and dicyemids in Bayesian analysis [7].

The probability of the independent emergence of *i* or more synapomorphies out of *n* possible ones was calculated via R 2.11.1 in the Rstudio version 1.0.136 using the frequency values of synopomorphies in the RefSeq database outside the study group (homoplasy frequencies) according to the following algorithm:

1. *h* = (*h_1_*, *h_2_*, …, *h_n_*_−*1*_, *h_n_*) # Each value of the vector corresponds to the homoplasy frequency of each synapomorphy according to the RefSeq database.
2. *w* = 1 − *h* # Each value of the vector corresponds to the probability of non-emergence of each synapomorphy.
3. *a_k_* = combn (*h*,*k*) # Matrices contain all cases of emergence of *k* synapomorphies, where *k* is a number from 1 to *n*.
4. *b_k_* = combn (*w*,*k*) # Matrices contain all cases of non-emergence of *k* synapomorphies, where *k* is a number from 1 to *n*.
5. *ν_k_* = apply (*a_k_*,2,prod) # The vector of probabilities of emergence of *k* synapomorphies for all possible subsets of *k* out of *n* synapomorphies.
6. *y_k_* = apply (*b_k_*,2,prod) # The vector of probabilities of non-emergence of *k* synapomorphies for all possible subsets of *k* out of *n* synapomorphies.
7. *p_k_* = *ν_k_* × *y_n_*_−*k*_ # The probabilities of independent emergence of exactly *k* out of *n* synapomorphies.
8. *q_k_* = sum (*p_k_*) # The probability of independent emergence of exactly *k* out of *n* synapomorphies.
9. *x_k_* = *q_k_* + *q_i_*_+*1*_ + … + *q_n_*_−*1*_ + *q_n_* # The total probability of independent emergence of *k* or more synapomorphies.

### 3.6. Probability of Loss of Synapomorphies

We hypothesized that, all else being equal, the probability of synapomorphies loss is higher in lineages with high evolution rates. The ratios of the relative rates of evolution of orthonectids and Annelida, Clitellata, Hirudinea and Siboglinidae were calculated by averaging distances from the common ancestor node to each leaf on the Bayesian tree and comparing it with the same value for orthonectids after placing them into the group, followed by re-estimating branch lengths (Table 9).

The relative evolutionary rate of dicyemids against a typical Annelida representative was also calculated and it was shown that the evolutionary rate of dicyemids is 21.57 times higher than that of annelids.

The probabilities of synapomorphies loss by orthonectids that were previously acquired from a common ancestor of each of the groups Annelida, Clitellata, Hirudinea and Siboglinidae (in the case of orthonectids belonging to these groups) were calculated in the Rstudio program using the frequency values of synopomorphies in this group according to the following algorithm:

1. *s* = (*s_1_*, *s_2_*, …, *s_n_*_−*1*_, *s_n_*) # Each value of vector corresponds to the frequency of occurrence of each synapomorphy in a given group.
2. *a* = (*a_i_*)*_,_ a_i_* = *s_i_*^*r* # The probabilities of remaining each synapomorphy in orthonectids, adjusted for the increased rate of evolution of orthonectids compared to the average representative of this group. Here *r* is a coefficient reflecting how much times the rate of evolution for orthonectids is higher than the rate of evolution for average representative of this group (see Table 1).
3. *b* = (*b_i_*), *b_i_* = 1 − *a_i_* # The probabilities of loss for each synapomorphy in orthonectids.
4. *p* = prod*_i_*_∈*M*_ (*b_i_*) # The *a priori* probability of loss for a specific subset *M* of synapomorphies in orthonectids.

## Figures and Tables

**Table 1:** Occurrence of Annelida synapomorphies in Metazoa according to the RefSeq and the selected thresholds.

Feature	Gene	Amino Acid Motif ^1^	Occurrence Among Annelida, %	Occurrence Outside Annelida, %
1	*atp6*	DGAPD-**W**-LNPFL	83	0.6
2	*atp6*	PLTLS-**F**-RLAAN	87	0.7
3	*cox1*	TAHAF-**L**-MIFFL	63	3.0
4	*cox1*	LSFVA-**L**-MLFIF	83	3.0
5	*cox1*	DPILP-**L**-DFHNL	81	1.3
6	*cox3*	TPEIG-**C**-SWPPT	97	1.4
7	*cox3*	VDVVW-**I**-CLYLC	92	2.7
8	*cytb*	EWYFL-**W**-MYAIL	94	0.1
9	*nad6*	VMFAY-**F**-LALTP	99	1.1

^1^ Amino acid motifs specified for *Lumbricus terrestris* (Accession Number NP_008242.1).

**Table 2:** Synapomorphies of Orthonectida and Annelida in predicted mitochondrial proteins.

Taxon	Protein, Amino Acid Motif ^1^ and Occurence, %			
	COX1	CYTB	ATP6	NAD6
	LSNMA-**L**-TLFMW	DWFLL-**W**-AYAIL	PLTLS-**F**-RICAI	SIYIF-**F**-ISSGG
Annelida	83	94	87	99
Basal branched annelids (*Owenia* + *Magelona* + Chaetopteridae)	0	0	0	33
Amphinomidae + Sipuncula	100	100	86	100
Crown annelids (Pleistoannelida)	94	97	96	100
Metazoa without Annelida	2.7	0.1	0.7	2.5

^1^ Amino acid motif specified for *Intoshia linei*.

**Table 3:** Average probabilities of independent emergence of annelid synapomorphies.

Number of Synapomorphies (*n*)	Average Probability of Independent Emergence of *n* Synapomorphies
1	0.014
2	2.2 × 10^−4^
3	3.13 × 10^−6^
4	4.13 × 10^−8^
5	4.95 × 10^−10^
6	5.23 × 10^−12^
7	4.87 × 10^−14^
8	3.78 × 10^−16^
9	2.04 × 10^−18^

**Table 4:** Number of synapomorphies with low level of homoplasy and high occurrence inside the group and their occurrence in orthonectids.

Taxon	Number of Species	Number of Taxon Synapomorphies (*n*)	Number of Taxon Synapomorphies in Orthonectids (*i*)
Annelida	157	9	4
Clitellata	65	12	3
Hirudinea	29	45	3
Siboglinidae	18	102	4

**Table 5:** Probabilities of independent emergence of *i* and more synapomorphies and probabilities of loss of *n*-*i* synapomorphies by orthonectids.

Taxon	Probability of Emergence	Probability of Loss
Annelida	1.9 × 10^−5^	0.33
Clitellata	0.0012	0.015
Hirudinea	0.014	2.3 × 10^−6^
Siboglinidae	0.017	3.3 × 10^−11^

**Table 6:** Occurrence of mesozoan synapomorphies according to the RefSeq and the chosen threshold.

Feature	Gene	Amino Acid Motif ^1^	Occurrence Outside Mesozoa, %
1	*atp6*	ILLFL-**Y**-DVMVC	1.4
2	*cox1*	LITAH-**G**-LIMIF	2.5
3	*cox1*	YLFSS-**S**-YSVDF	2
4	*cox1*	ISSIN-**S**-SINFF	2
5	*cox1*	HPEVY-**V**-LILPG	2
6	*cox1*	LFSQM-**S**-MIYAM	2.4
7	*cox1*	MIYAM-**G**-MIMFL	0.3
8	*cox1*	SMGAV-**Y**-LILGS	0.4
9	*cox1*	LLTLG-**S**-NMCFL	0.04
10	*cox2*	VLPYN-**K**-MCSLM	0.5
11	*cox3*	MLFFI-**F**-SEIMF	2.3
12	*cytb*	MLGLF-**M**-FLQTI	1.1
13	*cytb*	FYIQN-**E**-IFFGW	2.4
14	*cytb*	IGVSF-**I**-FILIY	0.8
15	*cytb*	MHMFR-**S**-IYFKL	1.3
16	*cytb*	LWFSG-**M**-LMFLL	0.8
17	*cytb*	LIIII-**S**-FLGYS	0.7
18	*cytb*	AKVIT-**S**-LFTII	3
19	*cytb*	LILWG-**D**-FTVAG	0.3
20	*cytb*	LWGDF-**T**-VAGPT	1
21	*cytb*	GSNNK-**F**-GLKNT	0.1
22	*cytb*	DSFME-**S**-NKLVT	1
23	*cytb*	MILLS-**Y**-CGGAI	1.9
24	*nd1*	FSFMT-**I**-LMSFY	2.4
25	*nd1*	FMMMI-**S**-FFSKS	0.9
26	*nd1*	MFWNF-**L**-LPIIL	2.2
27	*nd2*	NGFSS-**L**-FLFLS	2.4
28	*nd3*	SLKYF-**K**-IIMLF	0.1
29	*nd4*	LAHVE-**S**-PTEGS	0.5
30	*nd5*	AFFKS-**S**-LFLSF	0.4
31	*nd5*	FSKEK-**M**-FHMGY	0.9

^1^ Amino acid motifs specified for *Intoshia variabili*.

**Table 7:** Expected and observed number of potential synapomorphies in pairs between Orthonectida, Dicyemida, and Neodermata.

Groups	Expected Number of Features	Observed Number of Features	Observed/ Expected Ratio
Orthonectida+Dicyemida	7	22	3.1
Orthonectida+Neodermata	5	12	2.4
Dicyemida+Neodermata	6	15	2.5

**Table 8:** Occurrence of synapomorphies of *Lobatocerebrum* sp. and Mesozoa according to the RefSeq and the chosen threshold.

Feature	Gene	Amino Acid Motif ^1^	Mesozoan Groups with Feature	Occurrence Outside Mesozoa, %
1	*cox1*	SSYPF-**S**-SSYSM	Orthonectids	1.2
2	*cox1*	FTFGG-**F**-TGLYL	Orthonectids	0.3
3	*cox2*	YVLLE-**S**-CIIET	Both	0.9
4	*cox2*	VKLIA-**N**-QWFWT	Both	0.7
5	*cytb*	YSIHS-**V**-GVSII	Dicyemids	0.1
6	*nd1*	SEYIL-**M**-SIMAI	Orthonectids	2.3
7	*nd3*	FLLFN-**F**-LFLGL	Both	2.2
8	*nd3*	IFGLY-**Y**-ELGWG	Both	0.1
9	*nd4*	LFFYF-**K**-DMSMI	Orthonectids	0.6
10	*nd5*	IALST-**M**-NHLSI	Both	0.6

^1^ Amino acid motifs specified for *Intoshia variabili*.

**Table 9:** Relative rates of evolution of orthonectids and the average representative of the annelid taxonomic group.

Taxon	Average Distance from Common Ancestor	Ratio of Evolution Rates for Orthonectids and Individual Taxa
Annelida	0.87	18.83
Clitellata	0.44	37.23
Hirudinea	0.65	25.2
Siboglinidae	0.31	52.84

## Data Availability

The original contributions presented in this study are included in the article. Further inquiries can be directed to the corresponding authors.

## Supplementary Materials

The following supporting information can be downloaded at https://www.mdpi.com/article/10.3390/ijms26135983/s1.
