# Supplementary material for: Rare Evolutionary Events Support the Phylogenetic Placement of Orthonectida Within Annelida
Source: Int J Mol Sci. 2025 Jun 21;26(13):5983. doi: 10.3390/ijms26135983 (PMC12249979; doi:10.3390/ijms26135983)
Supplement: Supplementary file 1 [file ijms-26-05983-s001.zip › Figure S1.pdf]

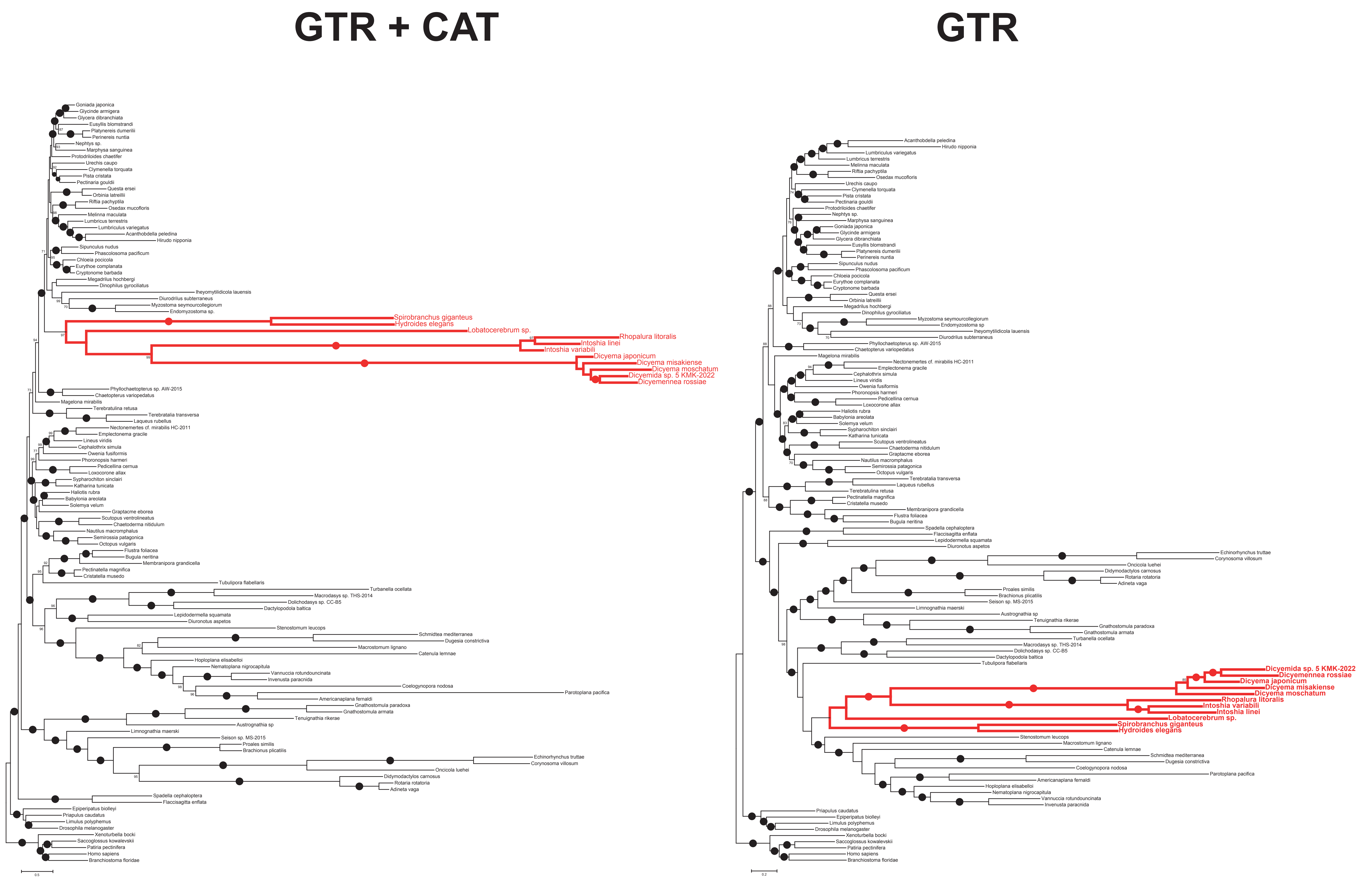

Figure S1: Bayesian trees reconstructed with the concatenated dataset of 12 mitochondrial proteins (a) under the GTR+CAT+Γ and (b) GTR+Γ models. Numbers at the branches indicate Bayesian posterior probabilities (in %), values below 70 are not shown, values of 100 are marked with dots.
