# Supplementary material for: Rare Evolutionary Events Support the Phylogenetic Placement of Orthonectida Within Annelida
Source: Int J Mol Sci. 2025 Jun 21;26(13):5983. doi: 10.3390/ijms26135983 (PMC12249979; doi:10.3390/ijms26135983)
Supplement: Supplementary file 1 [file ijms-26-05983-s001.zip › Figure S5.pdf]

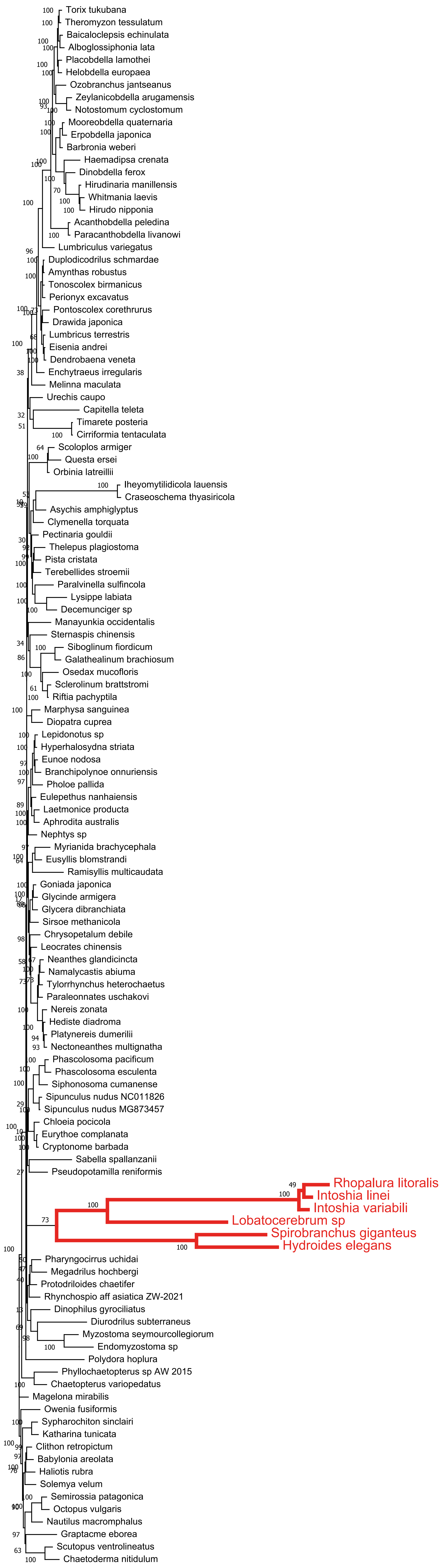

Figure S5: Bayesian tree based on initial alignment after removing the AT-rich codon coding amino acids (F, M, I, N, K) from orthonectid and lobatocerebrid sequences.
