# Supplementary material for: Rare Evolutionary Events Support the Phylogenetic Placement of Orthonectida Within Annelida
Source: Int J Mol Sci. 2025 Jun 21;26(13):5983. doi: 10.3390/ijms26135983 (PMC12249979; doi:10.3390/ijms26135983)
Supplement: Supplementary file 1 [file ijms-26-05983-s001.zip › Table S1.pdf]

Supplementary Materials

# Rare evolutionary events support the phylogenetic placement of Orthonectida within Annelida

**Olga V. Nikolaeva [1], Kirill V. Mikhailov [1,2], Maria S. Muntyan [1,*], Oleg A. Zverkov [2], Sergey A. Spirin [1,3,4], Vassily A. Lyubetsky [2], Georgy S. Slyusarev [5] and Vladimir V. Aleoshin [1,2,6*]**

1 Belozersky Institute of Physico-Chemical Biology, Lomonosov Moscow State University, Leninskie Gory, 119992 Moscow, Russia; olga_popova92@inbox.ru (O.V.N); kv.mikhailov@belozersky.msu.ru (K.V.M); sas@belozersky.msu.ru (S.A.S)
2 Institute for Information Transmission Problems (Kharkevich Institute), Russian Academy of Sciences, Bolshoy Karetny Per., 19, Bld. 1, Moscow 127051, Russia; zverkov@iitp.ru (O.A.Z.); lyubetsk@iitp.ru (V.A.L.)
3 Higher School of Economics, Myasnitskaya st., 20, Moscow 101000, Russia; sas@belozersky.msu.ru (S.A.S)
4 Federal State Institution "Scientific Research Institute for System Analysis of the Russian Academy of Sciences", Nakhimovsky prospect, 36, Bld. 1, Moscow 117218, Russia; sas@belozersky.msu.ru (S.A.S)
5 Department of Invertebrate Zoology, Faculty of Biology, Saint-Petersburg University, Universitetskaya emb. 7/9, 199034 St. Petersburg, Russia; slyusarev@hotmail.com (G.S.S.)
6 Faculty of Biology, Lomonosov Moscow State University, Leninskie Gory, 119234 Moscow, Russia; aleshin@genebee.msu.su (V.V.A.)
* Correspondence: muntyan@genebee.msu.ru (M.S.M.); aleshin@genebee.msu.su (V.V.A.)

**Table S1.** List of species used in phylogenetic analysis.

| Phylum/Subdivision | Species | Accession no. |
|---|---|---|
| Annelida: | | |
| basal brahcing annelids | *Owenia fusiformis* | NC_028712 |
| | *Magelona mirabilis* | NC_028711 |
| | *Chaetopterus variopedatus* | NC_028710 |
| | *Phyllochaetopterus* sp. | KT726961 |
| Amphinomidae | *Eurythoe complanata* | KT726962 |
| Sipuncula | *Phascolosoma pacificum* | NC_031412 |
| | *Sipunculus nudus* | NC_011826 |
| Lobatocerebridae | *Lobatocerebrum* sp.⋆ | SRX1122066 |
| Orthonectida | *Intoshia linei* | MG209116 |
| | *Intoshia variabili*⋆ | SRR8873482 |
| | *Rhopalura litoralis* | MG917727 |
| Myzostomida | *Endomyzostoma* sp. | FJ975144 |
| | *Myzostoma seymourcollegiorum* | EF506562 |
| | *Diurodrilus subterraneus* | KC790350 |
| Pleistoannelida | *Clymenella torquata* | NC_006321 |
| | *Eusyllis blomstrandi* | NC_031402 |
| | *Hirudo nipponia* | NC_023776 |
| | *Lumbricus terrestris* | NC_001673 |
| | *Marphysa sanguinea* | KF733802 |
| | *Megadrilus* sp.⋆ | SRX1026327 SRR2020581 |
| | *Nephtys* sp. | NC_010559 |
| | *Orbinia latreillii* | NC_007933 |
| | *Pista cristata* | NC_011011 |

| | | |
|---|---|---|
| | *Platynereis dumerilii* | NC_000931 |
| | *Protodriloides symbioticus*⋆ | SRX1116278 SRR2124791 SRR2124792 |
| | *Riftia pachyptila* | NC_026860 |
| | *Spirobranchus giganteus* | NC_032055 |
| | *Terebellides stroemii* | NC_011014 |
| | *Urechis caupo* | NC_006379 |
| Brachiopoda | *Laqueus rubellus* | NC_002322 |
| | *Phoronopsis harmeri* | NC_018761 |
| | *Terebratalia transversa* | NC_003086 |
| | *Terebratulina retusa* | NC_000941 |
| Bryozoa | *Bugula neritina* | NC_010197 |
| | *Flustra foliacea* | NC_016722 |
| | *Membranipora membranacea*⋆ | SRX1121923 SRR2131259 |
| | *Tubulipora flabellaris* | NC_015646 |
| Dicyemida | *Dicyema japonicum*⋆ | PRJDB4679 |
| | *Dicyema misakiense* | AB016262<br>AB016263<br>AB011832<br>AB011833<br>AB011834 |
| Entoprocta | *Loxocorone allax* | NC_010431 |
| | *Pedicellina cernua* | PRJNA280093 FJ196081 |
| Gastrotricha | *Diuronotus aspetos*⋆ | SRX1121926 SRR2131262 |
| | *Lepidodermella squamata* | NC_026985 |
| Gnathostomulida | *Austrognathia* sp.⋆ | SRX997150 SRR1976176 |
| | *Gnathostomula paradoxa* | NC_026984 |
| Micrognathozoa | *Limnognathia maerski*⋆ | SRX1121929 SRR2131287 |
| Mollusca | *Babylonia areolate* | NC_023080 |
| | *Chaetoderma nitidulum* | NC_013846 |
| | *Clithon retropictus* | NC_031893 |
| | *Graptacme eborea* | NC_006162 |
| | *Haliotis rubra* | NC_005940 |
| | *Katharina tunicata* | NC_001636 |
| | *Nautilus macromphalus* | NC_007980 |
| | *Octopus vulgaris* | NC_006353 |
| | *Scutopus ventrolineatus* | NC_025284 |
| | *Semirossia patagonica* | NC_016425 |
| | *Solemya velum* | NC_017612 |
| | *Sypharochiton sinclairi* | NC_024173 |
| Nemertea | *Cephalothrix linearis*⋆ | SRX534866<br>SRX534867<br>SRX534868 |
| | *Emplectonema gracile* | NC_016952 |
| | *Lineus viridis* | NC_012889 |
| Platyhelminthes | *Catenula lemnae*⋆ | SRX871445 SRR1796434 |
| | *Dugesia japonica* | NC_016439 |
| | *Hoploplana elisabelloi* | NC_028200 |
| | *Macrostomum lignano* | NC_035255 |
| | *Schmidtea mediterranea* | KM821047 |
| | *Stenostomum leucops*⋆ | SRX951992 SRR1910423 |

| | | |
|---|---|---|
| Syndermata | *Brachionus plicatilis* | NC_010472<br>NC_010484 |
| | *Oncicola luehei* | NC_016754 |
| | *Rotaria rotatoria* | NC_013568 |
| | *Seison* sp.⋆ | ERX219617<br>ERX219618 |
| outgroup: | | |
| Chaetognatha | *Spadella cephaloptera* | NC_006386 |
| Ecdysozoa | | |
| Priapulida | *Priapulus caudatus* | NC_008557 |
| Onychophora | *Epiperipatus biolleyi* | NC_009082 |
| Arthropoda | *Drosophila melanogaster* | KY310613 |
| | *Limulus polyphemus* | JX983598 |
| Deuterostomia | | |
| Cephalochordata | *Branchiostoma floridae* | NC_000834 |
| Vertrbrata | *Homo sapiens* | MF737176 |
| Echinodermata | *Patiria pectinifera* | NC_001627 |
| Hemichordata | *Saccoglossus kowalevskii* | NC_007438 |
| Xenacoelomorpha | *Xenoturbella bocki* | NC_008556 |

⋆ - sequences assembled in this study
