# Supplementary material for: Rare Evolutionary Events Support the Phylogenetic Placement of Orthonectida Within Annelida
Source: Int J Mol Sci. 2025 Jun 21;26(13):5983. doi: 10.3390/ijms26135983 (PMC12249979; doi:10.3390/ijms26135983)
Supplement: Supplementary file 1 [file ijms-26-05983-s001.zip › Table S2.pdf]

Table S2. Expected (first value) and observed (second value) number of pairwise homoplasies of nine annelid characters.

The observed value after combining species of the same genus is shown in parentheses. The numbers on the main diagonal show the frequencies (in %) of the corresponding homoplasies among Metazoa excluding Annelida according to the Refseq database. Color indicates cases where the observed number is significantly higher than the expected number.

1	0.6								
2	0|2	0.7							
3	0|0	0|0	3.1						
4	0|0	0|0	0|4	2.7					
5	0|0	0|0	0|0	0|0	0.6				
6	0|4	0|2	0|1	0|0	0|0	1.4			
7	1|4	1|7	1|4	1|0	0|0	3|52*	2.7		
8	0|3	0|0	0|0	0|0	0|0	0|3	0|3	0.1	
9	1|1	1|2	1|7	1|20*	0|0	1|20*	4|6	0|1	2.5
synapomorphy	1	2	3	4	5	6	7	8	9
