# Supplementary figures and images for: Rare Evolutionary Events Support the Phylogenetic Placement of Orthonectida Within Annelida
Source: Int J Mol Sci. 2025 Jun 21;26(13):5983. doi: 10.3390/ijms26135983 (PMC12249979; doi:10.3390/ijms26135983)

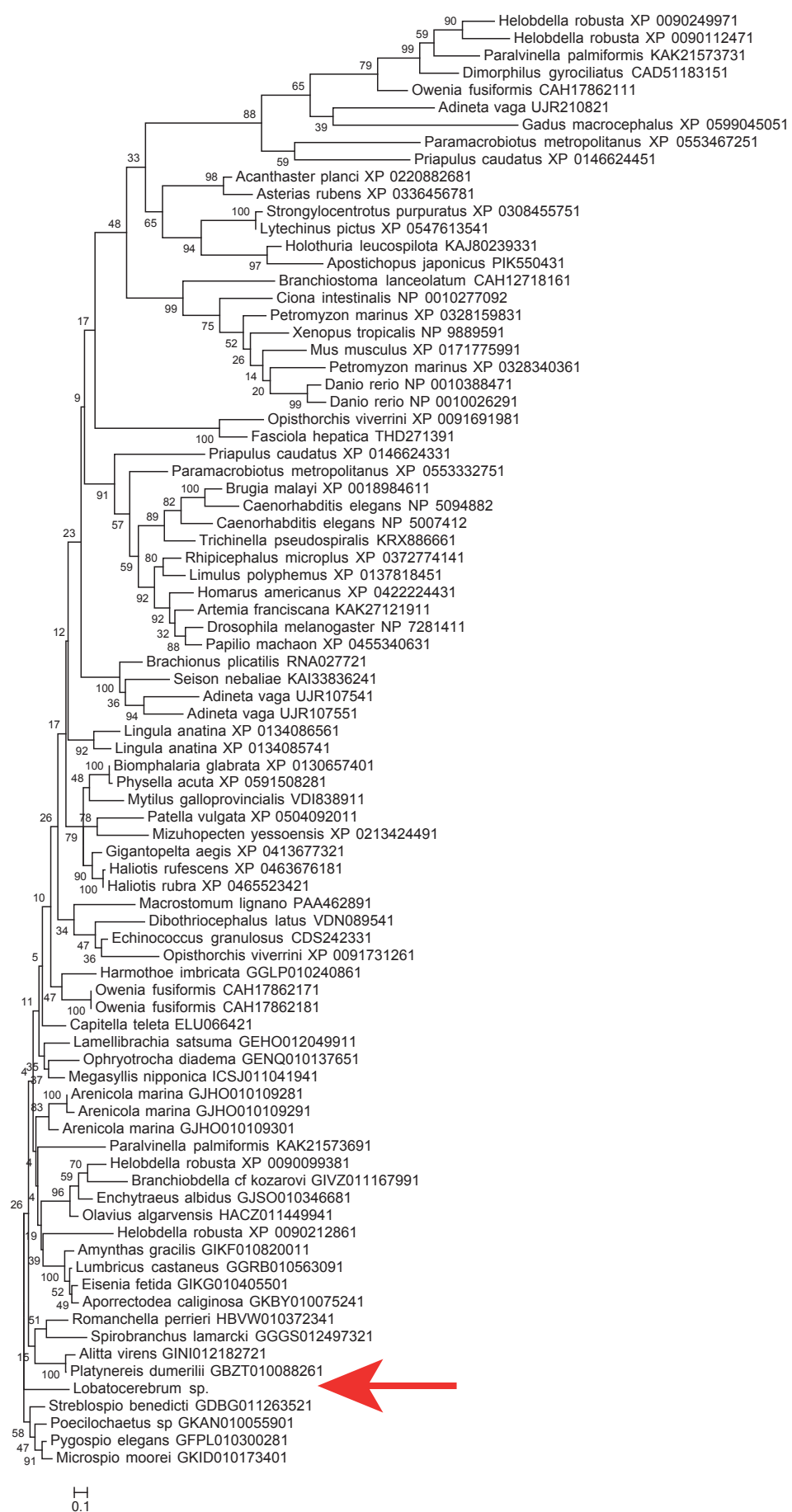

Figure S6: Bayesian tree based on troponin amino acid sequences.

Supplement: Supplementary file 1 [file ijms-26-05983-s001.zip › Figure S6.pdf]
